# Predictive Maintenance Scheduling with Failure Rate Described by Truncated Normal Distribution

**DOI:** 10.3390/s20236787

**Published:** 2020-11-27

**Authors:** Iwona Paprocka, Wojciech M. Kempa, Grzegorz Ćwikła

**Affiliations:** 1 Department of Engineering Processes Automation and Integrated Manufacturing Systems, Faculty of Mechanical Engineering, Silesian University of Technology, Konarskiego 18A str., 44-100 Gliwice, Poland; grzegorz.cwikla@polsl.pl; 2 Department of Mathematics Applications and Methods for Artificial Intelligence, Faculty of Applied Mathematics, Silesian University of Technology, 23, Kaszubska Str., 44-100 Gliwice, Poland; wojciech.kempa@polsl.pl

**Keywords:** predictive maintenance, production planning, MTTF, normal distribution, six sigma, reliability theory

## Abstract

The method of risk assessment and planning of technical inspections of machines and optimization of production tasks is the main focus of this study. Any unpredicted failure resulted in the production plans no longer being valid, production processes needing to be rescheduled, costs of unused machine production capacity and losses due to the production of poor-quality products increase, as well as additional costs of human resources, equipment, and materials used during the maintenance. The method reflects the operation of the production system and the nature of the disturbances, allowing for the estimation of unknown parameters related to machine reliability. The machine failure frequency was described with the normal distribution truncated to the positive half of the axis. In production practice, this distribution is commonly used to describe the phenomenon of irregularities. The presented method was an extension of the Six Sigma concept for monitoring and continuous control in order to eliminate and prevent various inconsistencies in processes and resulting products. Reliability characteristics were used to develop predictive schedules. Schedules were assessed using the criteria of solution and quality robustness. Estimation methods of parameters describing disturbances were compared for different job shop scheduling problems. The estimation method based on a maximum likelihood approach allowed for more accurate prediction of scheduling problems. The paper presents a practical example of the application of the proposed method for electric steering gears.

## 1. Introduction

Six Sigma is a popular technique used to eliminate and prevent inconsistencies in products, processes, and to describe and reduce process failures. For example, Erginel and Hasırcı [1] controlled the failure rate of the screwing backplate to the side panels of the product. They identified failure modes that affected the failure rate of the tightening process. They used the normal distribution and process knowledge to reduce the failure rate.

In line with Six Sigma to stabilize the process, all equipment must be in the best possible technical state, the equipment must be maintained, and the workforce must be trained in operating standards. One of the conditions for the efficient implementation of this methodology is the existence of a real-time data collection system, monitoring key points of the machine or device, where symptoms of impending problems can be detected. In the past, data on the condition of the machine during its operation were collected manually, mainly by the machine operator, using one’s subjective senses and experience, and communicated in the form of oral or written reports [2]. Today, devices can and should be equipped with a set of sensors monitoring both the state of the process (correct material feeding, technological parameters within the assumed range) and the machine itself (noise, vibrations, position of key machine elements, uniformity of movement, etc.). Machine condition checking sensors can be installed by the manufacturer in places provided by the machine designer or added later, in accordance with the selected scope of the machine and process condition monitoring. For machines equipped with an advanced control system, machine health and performance data can be collected directly from this control system, often equipped with standard interfaces. The data collection itself is only the initial stage of the process, during which the data should be filtered and interpreted in order to obtain more synthetic and easier to use information, allowing for operational decisions to be made. The issues of the systemic approach to data acquisition were analyzed, among others, in [3]. In order to stabilize the course of processes and minimize their variability, it was therefore necessary to use the existing sensors through the control system, installation of additional sensors (vibration sensors, accelerometers, etc.), and communication interfaces, as well as the implementation of appropriate data processing and integration procedures, along with convenient sharing of the results in a standardized form. The collected information can be used for online analyses during the operation of the device, as well as for the subsequent offline optimization of process parameters.

Given the data on the condition of the equipment and failure-free operation times, it is possible to describe the condition of the machine by the truncated normal distribution. The causes of possible deviations from the estimated time of failure-free operation are monitored. For the predictions to be reliable, it is necessary to use an appropriate method of estimating the parameters of the truncated normal distribution, which best describe the given failure phenomena [4]. With reliable normal distributions, one can begin the improvement process and the reduction of variation in the system. The presented method is thus an extension of the Six Sigma concept for monitoring and continuous control in order to eliminate and prevent inconsistencies in processes and resulting products. In this paper, three methods of estimating the parameters of the truncated normal distribution to describe the machine failure phenomenon were presented. Reliability of the estimation methods was compared for various job shop scheduling problems.

### 1.1. Complexity of Scheduling Problems

Production scheduling problems are considered difficult and important issues due to the high computational complexity. Computational complexity increases exponentially with the number of input data typical for a given problem. The complexity of the scheduling problem on parallel machines with sequence-dependent setup times depends on data such as the number of jobs, setup, and production operations or machines [5]. The complexity of the construction project scheduling problem increases with the growing amount of data on various labor productivity, adequacy of equipment, and weather conditions [6]. The problem of planning multi-process production with variable renewable integration depends on the model of energy consumption (energy sources), the number and size of buffers, parameters related to the production line and the production task [7]. The complexity of the berth scheduling problem at marine container terminals depends on the number and variability of input data on services and marine container number and routes [8]. The complexity also grows due to the multidimensionality of a solution space, which depends on a number of criteria adopted to evaluate schedules [5]. The high complexity of the problem causes the need to develop an effective algorithm, both in terms of computing time and power. Computing time and power demand can increase with the complexity of the planning problem, the number of procedures, and tuning parameters used in the algorithms. In addition, when planning production processes, not all input data is known in advance, e.g., machine unavailability times [9], renewable energy sources uncertainty [7], uncertainty of labor productivity, and weather conditions [6]. In the assumed planning horizon, there are disturbances (machine failures) that make the adopted schedule no longer valid. Determining the correct date for a machine inspection to protect the system from variation is still problematic. Too often performed preventive actions are treated as constraints in the production system and lead to a reduction of performance indicators. On the other hand, the machine may suddenly fail despite preventive maintenance, which implies the need to re-optimize the schedule.

### 1.2. Production and Maintenance Planning Practices

Machine maintenance reduces the frequency of unpredicted production stoppages. The tasks of maintenance personnel include following activities [10]: Corrective maintenance (CM), preventive maintenance (PM), and predictive maintenance (PdM). Corrective actions consist of repairing or replacing damaged machine elements [11]. Preventive actions consist of planning maintenance activities to prevent system failures [12] based on dynamic evaluation of components’ quality, degradation state, and the quality-reliability chain of a production system. Predictive activities may focus on “predicting the date of the next failure based on previous experiences instead of using online prediction” [13]. Better knowledge of the “nature” of disturbance and more reliable predictions of the downtime evolution over time are possible by analyzing historical data on the failure-free operation of components or machines.

Most studies in the field of scheduling production tasks and technical inspections of machines assume that machine inspection time or parameters describing the state of the machine are known at the beginning of the planning period [14,15,16]. The researchers ignore the variability aspect and focuses on the deterministic approach. Researchers also treat a machine inspection as a limitation of availability. However, the process of estimating the maintenance start time is stochastic. Each maintenance operation is scheduled for a specific time which can be described by fuzzy numbers and also depends on the impact on task completion dates [17]. Similarly, Cullum et al. [18] and Bali and Labdelaoui [19] consider the schedule of technical inspections as an optimization problem [18,19]. To select the appropriate inspection date, the cost function that describes the problem (e.g., inspection costs, repair costs, and risk) is minimized. However, it is difficult to quantify the cost of maintenance if the failure type and circumstances are not known. There are also planning methods that use the existing “time windows” to perform the technical inspection of the machine [20]. This approach is in line with the trend of eliminating the “muda”, but is not possible to apply for the bottleneck. Still, others introduce additional times (safety buffers) to the operation duration to minimize the impact of disruptions on a schedule [21]. This approach lowers the performance indicators of the production system. An interesting approach describes the state of a machine using a reliability function. Machine availability deteriorates at a predetermined maximum repair rate. Including the availability component in the objective function places a higher burden on assigning maintenance at intervals that increase the availability of machines. The best allocation of production and maintenance tasks for machines is searched in terms of criteria such as: Makespan or total lateness. After maintenance, the machine is restored to an “as new” [9] or “as bad as old” state [22]. Blokus and Kołowrocki determined a reliability function for an aging series system with the component dependency following the equal load-sharing rule [23]. Wand et al. proposed the reliability equation with three failure modes: Catastrophic (binary state) failure, degradation (continuous processes), and failure due to shocks (impulse processes). Two effects of shocks on performance were considered: A sudden increase in the failure rate and a direct random change in the degradation [24]. Kleiner et al. proposed a strategy for optimal rehabilitation of network pipes in which the water distribution network economics and hydraulic capacity were included. Deterioration of pipes was modeled using aging and stress causes [25]. Kołowrocki and Soszyńska–Budny proposed approaches for multistate systems with ageing components, changes to their structure, and their components’ reliability and safety parameters during the operation processes [26]. Neelacantan et al. modeled the deterioration of the quality of pipes as a result of aging. They noticed the relationship between the pipe diameter and the uptime and included it in the repair cost optimization model [27]. Romaniuk proposed a model for assessing the cost of maintaining the water supply network, where the quality and number of pipelines’ previous failures had a direct impact on reliability [28]. Song et al. analyzed multi-component systems with each component experiencing multiple failure processes due to exposure to degradation and shock loads. Each component of the model could fail and affect all components, potentially causing them to fail more frequently. In the model, the age replacement policy and an inspection-based maintenance policy were applied for the components. The optimal replacement interval or inspection times were searched in order to minimize a cost rate function [29].

Sakib and Wuest [30] noticed the popular practice for engineers and researchers to monitor historical data, model, and simulate using failure probabilities to predict failure-free time and system deterioration over the last decades. Following this spirit, the theory of probability has been proposed to support the data analysis for predictive inspections, machine condition, and remaining usage time or life-cycle management. Historical data on failure-free machine operation times is described by a normal distribution which is commonly used to describe parametric data. Most of the causes of variation are repetitive, and can thus be described by the truncated normal distribution. The normal distribution is symmetrical in relation to its expectation. It is therefore particularly suitable for modeling phenomena in which the probability of occurrence of values above and below the average is similar. For non-negative variables, the truncated normal distribution can be used. It allows for an unequal distribution of the probability mass below and above the expected value, and thus to obtain a distribution with right-hand asymmetry. This distribution, of course, remains a two-parameter one, which gives the possibility of its good fitting to the empirical data. The frequency of the machine failure is described by the normal distribution truncated to the positive half-axis as it describes changing the probability of failure with time.

### 1.3. Goals and Approaches

Compared to the analyzed literature on joint scheduling of production and maintenance tasks, the distinct nature of the work presented in this paper consists of combining the four: First, the truncated normal distribution was used to model the frequency of machine failures. More precisely, it was assumed that the successive failure-free times were distributed randomly according to the normal distribution truncated to the positive half-axis.

Second, this article is a response to the need to search for methods of estimating the parameters of the truncated normal distribution. Problems with the complementarity and credibility of historical data appeared. Therefore, the estimation method based on the reliability theory used only information about the number of disturbances in historical periods.

Third, the bottleneck is the machine that has the greatest impact on system capacity [31]. The predictive schedule served as an overall plan in the event of the bottleneck failure. The function of predictive planning was to guarantee stable and reliable operation of the production system in the event of disturbances. Thus, schedules were assessed using two criteria: Robustness of the solution and reliability of quality.

Fourth, a practical example of the application of the proposed maintenance planning method for an automotive company was provided.

Based on the review of reference publications, the following research points were identified:

- Methods of achieving the reliability parameters of the truncated normal distribution, even in the case of the absence of complementary and reliable data on historical failure-free times;
- Methods for obtaining the best maintenance and production schedules where the goal is to maximize stability and robustness.

The rest of the paper is organized as follows: The model of failures is presented in Section 2. Reliability characteristics for truncated normal distribution are described in Section 2.4. The maintenance and production scheduling method is described in Section 3. The job shop scheduling problem is described in Section 4. A comparison of the results of experimental tests of three estimation methods of the truncated normal distribution parameters is presented in Section 5. In Section 5.1, the example of estimation of reliability characteristics for the production line of electric steering gears is analyzed. The paper ends with a brief summary of the results and future research objectives (Section 6).

## 2. A Model of Failures

We considered a production planning model taking into account machine failure frequency. It was assumed that successive failure-fee times truncated normal distributions followed by repair times described by exponential distributions. It was assumed that the parameters of the distributions generally changed with time. Reliability characteristics predictions were built based on historical data on the number of machine failures and uptime in a certain number of historical periods of the same length. Such a system was monitored on *r* successive time periods, as can be seen in Equation (1):

(1) $$[0,\left.T\right),[T,\left.2T\right),\ldots ,\left[\left(r-1\right)T,rT\right)$$

of the same durations, for which the information about numbers of detected failures or failure-free times was known. The prediction of system behavior was built for the next period [*rT*, (*r* + 1)*T*]. We assumed that failure-free times *X_i_*_,1_,…,X*_i_*_,*Ni*_ in the *i*-th period [(*i* − 1)*T*, *iT*], *i* = 1, …, *r* + 1, had normal distributions with parameters m ∈ R and σ > 0, truncated to the positive half axis. The value *N_i_* represents the number of failures (being, in general, a random variable) detected in the *i*-th period. It is worth noting that the cumulative distribution function (CDF) *F*(⋅) of such a distribution has the Equation (2):

(2) $$F\left(t\right)=P\left\{X<t\left|X>0\right.\right\}=\frac{P\left\{0<X<t\right\}}{P\left\{X>0\right\}}=\frac{\Phi \left(\frac{t-m}{\sigma }\right)-\Phi \left(-\frac{m}{\sigma }\right)}{1-\Phi \left(-\frac{m}{\sigma }\right)},t>0,$$

where $\Phi \left(\cdot \right)$ stands for the CDF of the standard normal distribution and *X* is normally distributed with parameters m ∈ R and σ > 0. Differentiating the expression above with respect to variable *t*, we concluded that the probability density function (PDF) *f_i_*(⋅) of the arbitrary random variable *X_i_*_,*j*_, where *i* = 1, …, *N_i_*, had the following Equation (3):

(3) $$f_{i}\left(t\right)=\frac{1}{\sigma_{i}\left[1-\Phi \left(-\frac{m_{i}}{\sigma_{i}}\right)\right]}\phi \left(\frac{t-m_{i}}{\sigma_{i}}\right),t>0,$$

where $\phi \left(t\right)=\frac{1}{\sqrt{2\pi }}e^{-t^{2}/2}$ denotes the PDF of the standard normal distribution, and *m_i_* and *σ_i_* are parameters depending on the number of period *i*.

The mean value *E*(*X_i_*_,*k*_) and the variance *Var*(*X_i_*_,*k*_) of the normal distribution with parameters *m_i_* and *σ_i_* truncated to the positive half axis, with the PDF defined in (3), are given, respectively, by the following Equation (4):

(4) $$E\left(X_{i,k}\right)=m_{i}+\sigma_{i}\frac{\phi \left(-\frac{m_{i}}{\sigma_{i}}\right)}{1-\Phi \left(-\frac{m_{i}}{\sigma_{i}}\right)},$$

(5) $$Var\left(X_{i,k}\right)=\sigma_{i}^{2}\left[1-\frac{\frac{m_{i}}{\sigma_{i}}\phi \left(-\frac{m_{i}}{\sigma_{i}}\right)}{1-\Phi \left(-\frac{m_{i}}{\sigma_{i}}\right)}-{\left(\frac{\phi \left(-\frac{m_{i}}{\sigma_{i}}\right)}{1-\Phi \left(-\frac{m_{i}}{\sigma_{i}}\right)}\right)}^{2}\right],$$

where *k* = 1, …, *N_i_*.

At the end of reliable work period *X_i_*_,*k*_, as the failure occurs, a repair time *Y_i_*_,*k*_ begins immediately, and so on. Repair times *Y_i_*_,1_, …, *Y_i_*_,*Ni*_ for *i* = 1, …, *r* + 1, are supposed to be exponentially distributed with PDFs *g_i_*(⋅) as seen in Equation (6):

(6) $$g_{i}\left(t\right)=\left\{\begin{array}{l} \alpha_{i}\exp\left(-\alpha_{i}t\right),t>0,\\ 0,t\leq 0. \end{array}\right.$$

As it is well known [29]:

(7) $$E\left(Y_{i,k}\right)=\frac{1}{\alpha_{i}},$$

(8) $$Var\left(Y_{i,k}\right)=\frac{1}{\alpha_{i}^{2}},$$

where *σ_i_* > 0 is known for *i* = 1, …, *r* + 1, *E*(*Y_i_*_,*k*_)—the mean value of *Y_i_*_,*k*_, and *Var*(*Y_i_*_,*k*_)—the variance of *Y_i_*_,*k*_.

We took certain simplifying assumptiosn that each new period of the form [(*i* − 1)*T*, *iT*] started with the beginning of reliable work *X_i_*_,1_; in other words, we “deleted” the residual repair time *Y_i−_*_1,*Ni*_ in the *i*-th period [(*i* − 1)*T*, *iT*]. Thus, we can write:

(9) $$\sum_{k=1}^{N_{i}}Z_{i,k}=\sum_{k=1}^{N_{i}}\left(X_{i,k}+Y_{i,k}\right)\approx T,i=1,\ldots ,r+1.$$

Random variables *X_i_*_,*k*_, *Y_i_*_,*k*_, for *i* = 1, …, *r* + 1, and *k* = 1, …, *N_i_* are supposed to be totally independent. Thus, the evolution of the system can be observed on successive cycles *Z_i_*_,*k*_ = *X_i_*_,*k*_ + *Y_i_*_,*k*_, *i* = 1, …, *r* + 1, *k* = 1, …, *N_i_* which are independent random variables with PDFs defined as follows:

(10) $$h_{i}\left(t\right)=\int_{0}^{t}f_{i}\left(t-y\right)g_{i}\left(y\right)dy=\int_{0}^{t}g_{i}\left(t-y\right)f_{i}\left(y\right)dy=\frac{\alpha_{i}\exp\left(-\alpha_{i}t\right)}{\sqrt{2\pi }\alpha_{i}\left[1-\Phi \left(-\frac{m_{i}}{\sigma_{i}}\right)\right]}\int_{0}^{t}\exp\left[-\frac{{\left(y-m_{i}\right)}^{2}}{2\sigma_{i}^{2}}+\alpha_{i}y\right]dy\;=\frac{\alpha_{i}\exp\left(-\alpha_{i}t\right)}{\sqrt{2\pi }\alpha_{i}\left[1-\Phi \left(-\frac{m_{i}}{\sigma_{i}}\right)\right]}\int_{0}^{t}\exp\left[-\frac{{\left[y-\left(m_{i}+\sigma_{i}^{2}\alpha_{i}\right)\right]}^{2}}{2\sigma_{i}^{2}}+\frac{\sigma_{i}^{2}\alpha_{i}^{2}}{2}+m_{i}\alpha_{i}\right]dy\;=\frac{\alpha_{i}\exp\left(-\alpha_{i}t\right)}{\sqrt{2\pi }\alpha_{i}\left[1-\Phi \left(-\frac{m_{i}}{\sigma_{i}}\right)\right]}\int_{0}^{t}\exp\left[-\frac{{\left(y-m_{i}\right)}^{2}}{2\sigma_{i}^{2}}+\alpha_{i}y\right]dy$$

and CDFs as seen in Equation (11):

(11) $$H_{i}\left(t\right)=\int_{0}^{t}h_{i}\left(u\right)du=\frac{\alpha_{i}\exp\left[-\alpha_{i}\left(t-\frac{\alpha_{i}\sigma_{i}^{2}}{2}-m_{i}\right)\right]}{\sigma_{i}\left[1-\Phi \left(-\frac{m_{i}}{\sigma_{i}}\right)\right]}\int_{0}^{t}du\int_{0}^{u}\phi \left(\frac{y-\left(m_{i}+\alpha_{i}\sigma_{i}^{2}\right)}{\sigma_{i}}\right)dy.$$

### 2.1. Maximum Likelihood Approach

Suppose that in each of the intervals [0, *T*], [*T*, 2*T*], …, [(*r* − 1)*T*, *rT*], we have given sample values of random variables *X_i_*_,*k*_, *k* = 1, …, *N_i_* for any *i* = 1, …, *r* + 1, where *n_i_* is the observed value of *N_i_*. Thus, we have the following observations:

(12) $$\left(x_{1,1},x_{1,2},\ldots ,x_{1,n_{1}}\right),\ldots ,\left(x_{r,1},x_{r,2},\ldots ,x_{r,n_{r}}\right)$$

Let us consider the first historical period $\left[0,T\right)$. The maximum likelihood principle can be used to estimate parameters *m*_i_ and *σ*_i_ of truncated normal distribution as data *x*_1,1_, *x*_1,2_, …, *x*_1,*n*1_ which are independent and identically distributed random variables. We defined the likelihood function as follows [32,33]:

(13) $$\begin{array}{l} L\left(m_{1},\sigma_{1}\left|x_{1,1},x_{1,2}\ldots ,x_{1,n_{1}}\right.\right)=\prod_{k=1}^{n_{1}}f_{1}\left(x_{1,k}\right)=\prod_{k=1}^{n_{1}}\frac{1}{\sigma_{1}\left[1-\Phi \left(-\frac{m_{1}}{\sigma_{1}}\right)\right]}\phi \left(\frac{x_{1,k}-m_{1}}{\sigma_{1}}\right)=\\ =\frac{1}{{\left\{\sqrt{2\pi \sigma_{1}\left[1-\Phi \left(-\frac{m_{1}}{\sigma_{1}}\right)\right]}\right\}}^{n_{1}}}\exp\left[-\frac{1}{2\sigma_{1}^{2}}\sum_{k=1}^{n_{1}}{\left(x_{1,k}-m_{1}\right)}^{2}\right]. \end{array}$$

From Equation (13), we obtain:

(14) $$\ln L\left(m_{1},\sigma_{1}\left|x_{1,1},x_{1,2}\ldots ,x_{1,n_{1}}\right.\right)=-n_{1}\left\{\ln\sigma_{1}+\ln\left[1-\Phi \left(-\frac{m_{1}}{\sigma_{1}}\right)\right]+\frac{1}{2}\ln2\pi \right\}-\frac{1}{2\sigma_{1}^{2}}\sum_{k=1}^{n_{1}}{\left(x_{1,k}-m_{1}\right)}^{2}$$

Now, differentiating on variables *m*_1_ and *σ*_1_, we obtain the following system of normal equations:

(15) $$-\frac{n_{1}}{\sigma_{1}\left[1-\Phi \left(-\frac{m_{1}}{\sigma_{1}}\right)\right]}\phi \left(-\frac{m_{1}}{\sigma_{1}}\right)+\frac{1}{\sigma_{1}^{2}}\sum_{k=1}^{n_{1}}\left(x_{1,k}-m_{1}\right)=0,$$

(16) $$-\frac{n_{1}}{\sigma_{1}}+\frac{n_{1}m_{1}}{\sigma_{1}^{2}\left[1-\Phi \left(-\frac{m_{1}}{\sigma_{1}}\right)\right]}\phi \left(-\frac{m_{1}}{\sigma_{1}}\right)+\frac{1}{\sigma_{1}^{3}}\sum_{k=1}^{n_{1}}{\left(x_{1,k}-m_{1}\right)}^{2}=0.$$

Substituting Equation (15) into Equation (16), we obtain:

(17) $$-\frac{n_{1}}{\sigma_{1}}+\frac{m_{1}}{\sigma_{1}}\cdot \frac{1}{\sigma_{1}^{2}}\sum_{k=1}^{n_{1}}\left(x_{1,k}-m_{1}\right)+\frac{1}{\sigma_{1}^{3}}\sum_{k=1}^{n_{1}}{\left(x_{1,k}-m_{1}\right)}^{2}=0.$$

and hence:

(18) $$\sigma_{1}^{2}=\frac{m_{1}\sum_{k=1}^{n_{1}}\left(x_{1,k}-m_{1}\right)+\sum_{k=1}^{n_{1}}{\left(x_{1,k}-m_{1}\right)}^{2}}{n_{1}}=\frac{\sum_{k=1}^{n_{1}}x_{1,k}^{2}-m_{1}\sum_{k=1}^{n_{1}}x_{1,k}}{n_{1}}.$$

Now, multiplying Equation (15) by $\sigma_{1}^{2}\left[1-\Phi \left(-\frac{m_{1}}{\sigma_{1}}\right)\right]$ we obtain:

(19) $$-n_{1}\sigma_{1}\phi \left(-\frac{m_{1}}{\sigma_{1}}\right)+\left[1-\Phi \left(-\frac{m_{1}}{\sigma_{1}}\right)\right]\sum_{k=1}^{n_{1}}\left(x_{1,k}-m_{1}\right)=0.$$

Introducing *σ*_1_ given by Equation (18) into Equation (19) we eliminated *m*_1_ numerically, using one of the approximations of the cumulative distribution function and the probability density function of the standard normal distribution. Indeed, using the Maclaurin expansion, we obtain:

(20) $$\phi \left(x\right)\approx \frac{1}{\sqrt{2\pi }}\left(1-\frac{x^{2}}{2}+\frac{x^{4}}{8}\right).$$

Similarly, in [34] the following logistic approximation for Φ(*x*) was proposed:

(21) $$\Phi \left(x\right)\approx \frac{1}{1+e^{-1.702\cdot x}}.$$

After finding estimators ${\hat{m}}_{1},\ldots ,{\hat{m}}_{r}$ and ${\hat{\sigma }}_{1},\ldots ,{\hat{\sigma }}_{r}$ we extrapolated values ${\hat{m}}_{r+1}$ and ${\hat{\sigma }}_{r+1}$ for the period [*rT*, (*r* + 1)*T*] for which we had no observation, using the regression method.

### 2.2. Empirical Moments Approach

Suppose that we had given sample values *x*_1,1_, *x*_1,2_, …, *x*_1,*n*1_ for period [0, *T*] as in the previous section. We introduced the sample mean and the variance as follows [32,33]:

(22) $${\bar{x}}_{1}=\frac{1}{n_{1}}\sum_{k=1}^{n_{1}}x_{1,k},s^{2}\left(x_{1}\right)=\frac{1}{n_{1}}\sum_{k=1}^{n_{1}}{\left(x_{1,k}-{\bar{x}}_{1}\right)}^{2}.$$

Comparing empirical moments to theoretical ones (see Equations (4) and (5)), we obtained the following system of equations:

(23) $${\bar{x}}_{1}=m_{1}+\sigma_{1}\frac{\phi \left(-\frac{m_{1}}{\sigma_{1}}\right)}{1-\Phi \left(-\frac{m_{1}}{\sigma_{1}}\right)}$$

and

(24) $$s^{2}\left(x_{1}\right)=\sigma_{1}^{2}\left[1-\frac{\frac{m_{1}}{\sigma_{1}}\phi \left(-\frac{m_{1}}{\sigma_{1}}\right)}{1-\Phi \left(-\frac{m_{1}}{\sigma_{1}}\right)}-{\left(\frac{\phi \left(-\frac{m_{1}}{\sigma_{1}}\right)}{1-\Phi \left(-\frac{m_{1}}{\sigma_{1}}\right)}\right)}^{2}\right]$$

Using the same approximation (see Equations (20) and (21)), we found estimators for ${\hat{m}}_{1}$ and ${\hat{\sigma }}_{1}$ from Equations (23) and (24). After finding estimators ${\hat{m}}_{1},\ldots ,{\hat{m}}_{r}$ and ${\hat{\sigma }}_{1},\ldots ,{\hat{\sigma }}_{r}$ we built predictions for values ${\hat{m}}_{r+1}$ and ${\hat{\sigma }}_{r+1}$ using the regression.

### 2.3. Renewal Theory Approach

The last proposed method was based on the renewal theory. Recall that if ξ_1_, ξ_2_,… were nonnegative and independent random variables with the same distribution function *B*(*t*); then the following stochastic process:

(25) $$\nu \left(t\right)=\max\left\{n\in N:\sum_{i=1}^{n}\xi_{i}\leq t\right\},t\geq 0$$

is called a renewal process generated by random variables ξ_1_, ξ_2_,… with renewal moments $t_{n}=\sum_{i=1}^{n}\xi_{i},n=1,2,\ldots$

Following the properties of renewal process, we expressed the first and the second moments of *v*(*t*) by means of convolutions of distribution function *H*_1_(*t*) [34]; thus, we obtained:

(26) $$E\nu \left(t\right)=\sum_{n=1}^{\infty }H_{1}^{n*}\left(t\right)$$

(27) $$E\nu^{2}\left(t\right)=2\sum_{n=1}^{\infty }nH_{1}^{n*}\left(t\right)-\sum_{n=1}^{\infty }H_{1}^{n*}\left(t\right),$$

where successive convolutions $H_{1}^{n*}\left(t\right)$ are defined as follows:

(28) $$H_{1}^{1*}\left(t\right)=H_{1}\left(t\right),H_{1}^{n*}\left(t\right)=\int_{0}^{t}H_{1}^{\left(n-1\right)*}\left(t-y\right)dH_{1}\left(y\right),n\geq 2.$$

We introduced Laplace–Stieltjes transforms of appropriate functions in the following way:

(29) $$\Omega_{1}\left(s\right)=\int_{0}^{\infty }e^{-st}dE\nu \left(t\right),\Omega_{2}\left(s\right)=\int_{0}^{\infty }e^{-st}dE\nu^{2}\left(t\right),$$

(30) H1^s=∫0∞e−stdH1t,Re(s)>0

Since the transform of convolution equals the product of transforms, then we obtained from Equation (23):

(31) $$\Omega_{1}\left(s\right)=\int_{0}^{\infty }e^{-st}d\left(\sum_{n=1}^{\infty }H_{1}^{n*}\left(t\right)\right)=\sum_{n=1}^{\infty }{\left({\hat{H}}_{1}\left(s\right)\right)}^{n}=\frac{{\hat{H}}_{1}\left(s\right)}{1-{\hat{H}}_{1}\left(s\right)},$$

since $\left|\hat{H_{1}}\left(s\right)\right|<1.$ Similarly we can prove that:

(32) $$\Omega_{2}\left(s\right)=\frac{{\hat{H}}_{1}\left(s\right)-{\left[{\hat{H}}_{1}\left(s\right)\right]}^{2}}{{\left[1-{\hat{H}}_{1}\left(s\right)\right]}^{2}}.$$

Since $\hat{H_{1}}\left(s\right)$ can be calculated numerically as a function of unknown parameters *m*_1_ and *σ*_1_, we used a method of the Laplace or Laplace–Stieltjes in order to invert the right sides of Equations (27) and (28) on argument s. The right sides in Equations (27) and (28) are described by *R*_1_(t, *m*_1_, *σ*_1_) and *R*_2_(t, *m*_1_, *σ*_1_), respectively.

We can compare them to the following empirical estimators of *Eν*(*t*) and *Eν ^2^*(*t*), respectively:

(33) $$n_{1},s^{2}+n_{1}^{2}$$

where $s^{2}=\frac{1}{r}\sum_{i=1}^{r}{\left(n_{i}-\bar{n}\right)}^{2}$ and *n*_1_, …, *n*_r_ are numbers of failures physically observed in successive periods [0, *T*], [*T*, 2*T*], …, [(*r* − 1)*T*, *rT*] and $\bar{n}=\frac{1}{r}\sum_{i=1}^{r}n_{i}.$ From the system of equations:

(34) $$n_{1}=R_{1}\left(t,m_{1},\sigma_{1}\right),s^{2}+n_{1}^{2}=R_{2}\left(t,m_{1},\sigma_{1}\right)$$

we estimate unknown parameters *m*_1_ and *σ*_1_.

For the next period [*T*, 2*T*], we used the same algorithm for parameters *m*_2_ and *σ*_2_ with *n*_2_ instead of *n*_1_ as the first estimator in Equation (29). Using ${\hat{m}}_{1},\ldots ,{\hat{m}}_{r}$ and ${\hat{\sigma }}_{1},\ldots ,{\hat{\sigma }}_{r}$ we found predictions for values ${\hat{m}}_{r+1}$ and ${\hat{\sigma }}_{r+1}$ using the classical regression.

### 2.4. Predictions of Reliability Characteristics

Consider the interval [(*r* − 1)*T*, *rT*] for which estimators (using one of three methods) ${\hat{m}}_{r+1}$ and ${\hat{\sigma }}_{r+1}$ of parameters for truncated normal distribution were achieved. Below are formulas for the reliability characteristics used in the presented model:

The reliability function *R*(*t*), [(*r* − 1)*T*, *rT*] gives the probability that, beginning with moment *t*_0_ = *rT*, the first failure occurs after time *t*:

(35) $$R\left(t\right)=P\left\{X_{r+1,1}>t\right\}=\int_{t}^{\infty }f_{r+1}\left(u\right)du=\frac{1}{\sigma_{r+1}\left[1-\Phi \left(-\frac{m_{r+1}}{\sigma_{r+1}}\right)\right]}\int_{t}^{\infty }\phi \left(\frac{u-m_{r+1}}{\sigma_{r+1}}\right)du.$$

The mean time to first failure (MTTFF) is as follows:

(36) $$MTTFF=\int_{t}^{\infty }R\left(t\right)dt=\frac{1}{\sigma_{r+1}\left[1-\Phi \left(-\frac{m_{r+1}}{\sigma_{r+1}}\right)\right]}\int_{0}^{\infty }dt\int_{t}^{\infty }\phi \left(\frac{u-m_{r+1}}{\sigma_{r+1}}\right)du.$$

The mean time between failures equals mean time to failure plus mean time of repair, or, MTBF = MTTF + MTTR:

(37) $$E\left[X_{r+1,1}+Y_{r+1,1}\right]=m_{r+1}+\sigma_{r+1}\frac{\phi \left(-\frac{m_{r+1}}{\sigma_{r+1}}\right)}{1-\Phi \left(-\frac{m_{r+1}}{\sigma_{r+1}}\right)}+\frac{1}{\alpha_{r+1}}.$$

Probability *P* indicates that in the interval [*a*, *b*] ∈ [*rT*, (*r* + 1)*T*] there occurs at least one failure:

(38) $$p=P\left\{a\leq X_{r+1,1}\leq b\right\}=\int_{a}^{b}f_{r+1}\left(u\right)du=\frac{1}{\sigma_{r+1}\left[1-\Phi \left(-\frac{m_{r+1}}{\sigma_{r+1}}\right)\right]}\int_{a}^{b}\phi \left(\frac{u-m_{r+1}}{\sigma_{r+1}}\right)du.$$

**Figure 1:**
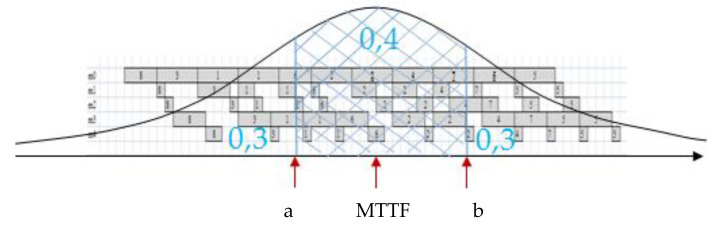
The period of increased risk of failure [*a*, *b* + mean time of repair (*MTTR*)] for the truncated normal distribution.

The period of increased probability of failure [*a*, *b* + *MTTR*] (Figure 1), where *a* is estimated based on the assumption that the probability of the failure-free time of the machine is higher than a equals 30%, and *b* is estimated based on the assumption that the probability of the failure-free time of the machine is less than b equals 70%:

(39) $$\begin{array}{l} P\left\{X_{r+1,1}>a\right\}=0,7\to 1-F_{x}\left(a\right)=0,7\to F_{x}\left(a\right)=0,3\\ \frac{\Phi \left(\frac{a-m_{r+1}}{\sigma_{r+1}}\right)-\Phi \left(-\frac{m_{r+1}}{\sigma_{r+1}}\right)}{1-\Phi \left(-\frac{m_{r+1}}{\sigma_{r+1}}\right)}=0,3. \end{array}$$

(40) $$\begin{array}{l} P\left\{X_{r+1,1}<b\right\}=0,7\to F_{x}\left(b\right)=0,7\\ \frac{\Phi \left(\frac{b-m_{r+1}}{\sigma_{r+1}}\right)-\Phi \left(-\frac{m_{r+1}}{\sigma_{r+1}}\right)}{1-\Phi \left(-\frac{m_{r+1}}{\sigma_{r+1}}\right)}=0,7. \end{array}$$

The obtained reliability characteristics were used in building a robust schedule *u*, *u* = 1,…,*w*. The accuracy of the prediction was computed for the assumption that *X_i_*_,1_, …, *X_i_*_,*Ni*_ had normal distributions with parameters *m* ∈ R and σ > 0, truncated to the positive half-axis and estimated using the described approaches (Section 2.1, Section 2.2 and Section 2.3):

(41) $$\Psi \left(u\right)=\left|\frac{X_{r+1,1}-MTTF}{X_{r+1,1}}\right|$$

The *MTTF* is *E*[*X_r_*_+1,1_] (37). The prediction of *MTTF* was effective if ex-post error was less than 0.05.

$$\Psi \left(u\right)\leq 0.05$$

The impact of changing real failure times X_r+1,1_ on the stability and robustness criteria was investigated for robust schedule *u*. Different values of reliability predictions achieved by the described approaches were used to generate schedules. Reliability parameters that guarantee the achievement of the most stable and robust schedules in the event of disruptions were adopted.

## 3. A Predictive Scheduling Model of Production and Maintenance

The process of building stable and robust schedules begins with generating a basic schedule. The scheduling problem relates to the allocation of production jobs to a limited number of machines. Having the basic schedule, the critical machine was identified. The critical machine was the one which was the most heavily loaded in the period described by reliability characteristics [*a*, *b* + *MTTR*]. Basic schedules were obtained using an immune algorithm [35]. Supporters of artificial intelligence methods added the ability to use information to improve the effectiveness of a solution search process. For supporters of the “non-free lunch theorem”, the proposed procedure returned schedules robust to specific types of disturbances, which better took into account the specificity of the processes under consideration. Knowledge gained about reliability characteristics improved the searching process of solutions, which was based not only on the “generate, modify, and test” principle. The best basic schedule was selected for the minimal value of the function:

(42) $$FF\left(u\right)=\varpi_{1}\cdot C\left(u\right)+\varpi_{2}\cdot F\left(u\right)+\varpi_{3}\cdot T\left(u\right)+\varpi_{4}\cdot I\left(u\right),$$

where: ϖ1, …, ϖ4 are weights of criteria, defined by a decision maker, ϖ1, ϖ2, ϖ3, ϖ4 ∈[0,1] and sum of weights is equal to 1.

*C*(*u*) is the makespan function:

(43) $$C\left(u\right)=\max\left[t_{z,V_{j}}\right],$$

*F*(*u*) is the total flow time of jobs:

(44) $$F\left(u\right)=\sum_{j=1}^{J}\left(t_{z,V_{j}}-t_{r,1_{j}}\right),$$

*T*(*u*) is the total delay of jobs:

(45) $$T\left(u\right)=\sum_{j=1}^{J}\left[0,D_{j}\right],where\left\{\begin{array}{l} 0,\;if\;d_{j}-t_{z,V_{j}}\leq 0\\ D_{j},\;if\;d_{j}-t_{z,V_{j}}>0, \end{array}\right.$$

*I* (*u*) is the idle time of machines:

(46) $$I\left(u\right)=\sum_{l=1}^{ł}I_{l},$$

where *t*_z,vj_ is the end time of operation *v*_j_ of job *j*, *v*_j_ = 1, …, *V_j_*, *j* = 1, …, *J*, t_r,vj_ is the start time of operation *v*_j_ of task *j*, *d_j_* is the deadline of job *j*, *D_j_* is delay in completing job *j*, and *I_l_* is the idle time of machine *l*, *l* = 1,…,*L*.

The predictive schedule was built by modifying the basic schedule using a heuristic: The minimal impact of disrupted operations on the schedule (*MIDOS*) [35]. According to the heuristic, a technical inspection of a machine was planned for the period described by [*MTTF*, *MTTF* + *MTTR*]. In the period of high risk of the machine failure [*a*, *b* + *MTTR*], the most flexible operations were assigned. An operation flexibility depended on: (1) The number of changes made in the event of an operation disruption ${\hat{\nu }}_{j}$ performed on the critical machine, which equals the number of operations to be rescheduled, $R_{{\hat{v}}_{j}}$, (2) the number of parallel machines in which the disrupted operation ${\hat{\nu }}_{j}$ can be performed alternatively, $F_{{\hat{v}}_{j}}$:

(47) $$MIDOS_{{\hat{v}}_{j}}=R_{{\hat{v}}_{j}}+\left(L-F_{{\hat{v}}_{j}}\right)\to \min,$$

where *L* is the number of machines.

The reactive schedule was generated in a situation where the predictive schedule needed to be updated after the disturbance. The newly generated schedule should reproduce as much as possible the previous one by introducing changes caused by the delay of the job disturbed by the machine failure. The stability criterion evaluated the reproducibility of schedule *u*:

(48) $$SR\left(u*\right)=\sum_{j=1}^{J}\sum_{v_{j}=1}^{V_{j}}\left|st_{j,v_{j}}\left(u\right)-st_{j,v_{j}}\left(u*\right)\right|$$

where $st_{j,v_{j}}\left(u\right)$ is start time of operation *v_j_* of job *j* in predictive schedule *u*; $st_{j,v_{j}}\left(u*\right)$ is the start time of operation *v_j_* of job *j* in reactive schedule u*;

After the machine failure, the value of the criterion used to assess the predictive schedule should not deteriorate significantly. The robustness of schedule *u* is assessed by:

(49) $$QR\left(u^{*}\right)=\left|FF\left(u\right)-FF\left(u^{*}\right)\right|,$$

where:

(50) $$FF\left(u*\right)=\varpi_{1}\cdot C\left(u*\right)+\varpi_{2}\cdot F\left(u*\right)+\varpi_{3}\cdot D\left(u*\right)+\varpi_{4}\cdot I\left(u*\right)$$

Reactive schedules u* were generated using heuristics: (1) Right shifting (RSh), and (2) re-scheduling operations on the first available parallel machine (RDO). The best schedule was selected using the rule: The minimal impact of rescheduled operations on schedule *u* (*MIROS*). Robustness QR(u*) and stability *SR*(u*) of reactive schedule u* were computed in the *MIROS* rule:

(51) MIROSu*=0.5QRu*QRu**+0.5SRu*SRu**,

where *QR*(u**)—the maximum value achieved for the quality robustness criterion, and *SR*(u**)—the maximum value achieved for the solution robustness criterion.

To sum up, the three stages could be distinguished in the process of developing robust and stable schedules: The construction of basic, predictive, and reactive schedules. The predictive schedules achieved were possible trade-offs between two criteria: Stability (Equation (49)) and robustness (Equation (51)).

## 4. Mean Time to Failure MTTF Prediction

Computer simulations are used for job shop scheduling problems. Jobs were processed in the exclude-like mode, and operations were non-preemptive. In addition, the job shop system was described by failure-free times of the critical machine for a number of historical periods (horizons) (Table 1). The duration of each scheduling horizon *i* equaled 1000 h. Repair times were described by exponential distributions with given parameters α_i_ in period *i*. Suppose the predicted value of α_36_ was 0.5, the expected distribution value (the mean repair time) was 2 h. After the machine failure, the rescheduled operations could be performed on parallel machines.

**Figure 2:**
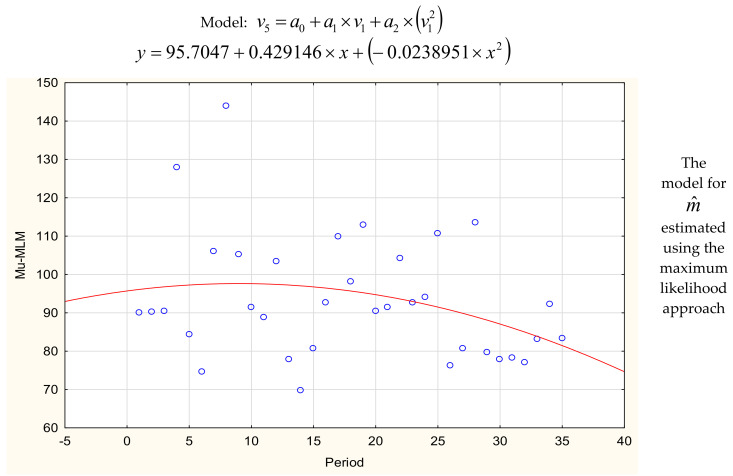

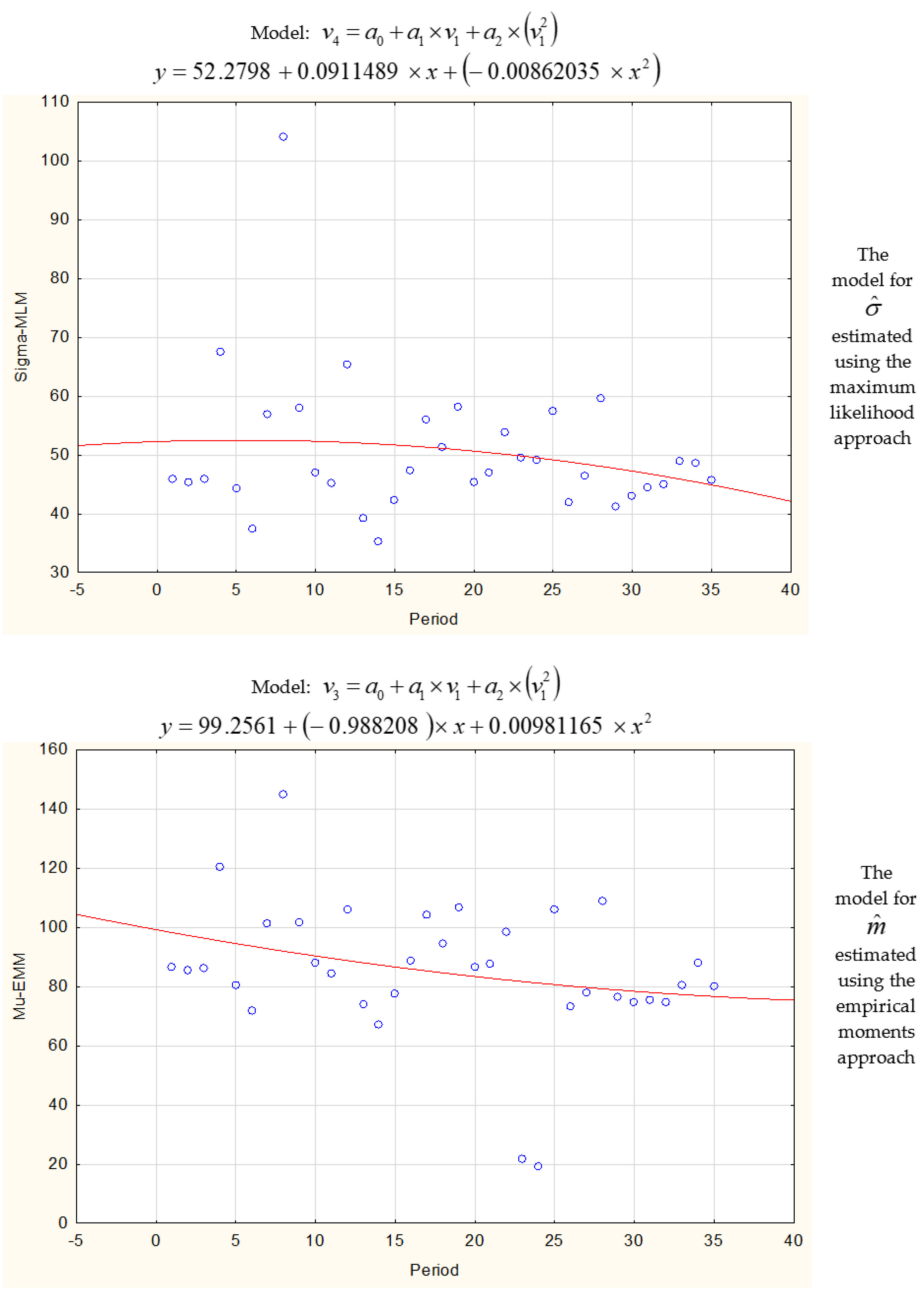

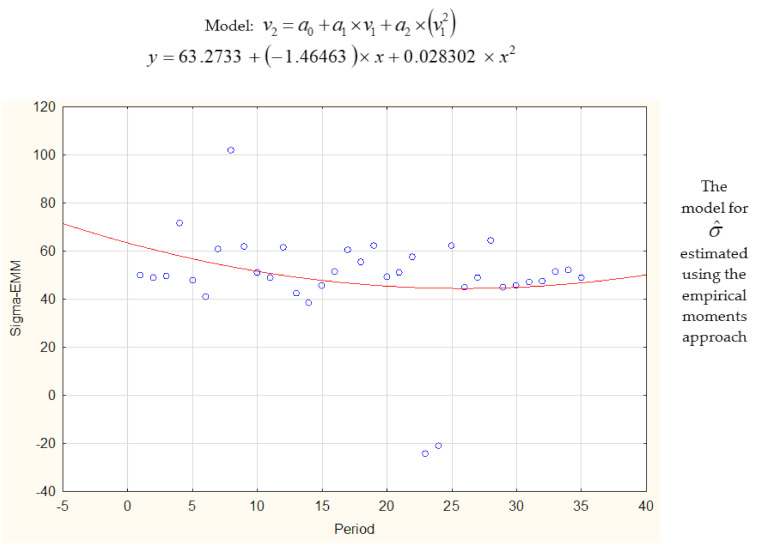
The models describing parameters $\hat{\sigma }$, $\hat{m}$ achieved using the Gauss–Newton method and the fitted functions.

Parameters *m*_1_, …, *m*_35_ and *σ*_1_, …, *σ*_35_ of normal distributions truncated to the positive half-axis described the failure-free times. First, the parameters were estimated using the maximum likelihood method. Parameter σ_1_ was numerically estimated using Equation (18) and parameter *m*_1_ was estimated using Equations (19)–(21). After finding successive estimators *m*_1_, …, *m*_35_ and *σ*_1_, …, *σ*_35_, values of ${\hat{m}}_{36}$ and ${\hat{\sigma }}_{36}$ were estimated for the future period [35*T*, 36*T*] using the least squares method or Gauss–Newton method (Table 2). The estimated models were described using the significance test for coefficients, standard error, R^2^, value of coefficient belonging to the interval with probability 95%, and *p*-value in Table 3. Values of estimators *m*_1_, …, *m*_35_ and *σ*_1_, …, *σ*_35_ together with the fitted functions are plotted in Figure 2.

The expected value (EX) of normal distributions truncated to the positive half-axis described by parameters achieved using the maximum likelihood method and least squares estimation method was 93.11. The increased probability of the machine failure was indicated at the time period [*a*, *b* + *MTTR*]. Applying Equations (39) and (40), parameters a = 58 and b = 102.28 were achieved.

Next, parameters *m*_1_, …, *m*_35_ and *σ*_1_, …, *σ*_35_ were computed using the formulas achieved for the empirical moments approach (Equations (23) and (24)). The functions describing *m*_1_, …, *m*_35_ and *σ*_1_, …, *σ*_35_ are presented in Table 2 and Figure 2. The predicted values of ${\hat{m}}_{36}$ and ${\hat{\sigma }}_{36}$ were equal 74.95 and 45.92 for the horizon [35*T*, 36*T*]. Mean time to failure was *EX* = 88.69. The increased probability of the machine failure was indicated at the time window [23, 125 + *MTTR*] (Equations (39) and (40)).

Experimental tests were carried out for job shop scheduling systems of different sizes in order to assess the accuracy of achieved values of reliability parameters. Each experiment was described by the number of jobs and the number of machines: (11 × 10), (10 × 9), (9 × 8), (8 × 7), and (7 × 6). Processes could have different machine routes, operation times, and deadlines in the experiments. The first machine was critical. Two sets of reliability predictions—*MTTF*, *MTTR*, *a* and *b*—were calculated using both approaches: Maximum likelihood and sample moments. Experiments were run to answer the question of what the stability and robustness of predictive schedules would be if the actual failure-free time of the critical machine X_36,1_ was equal to 80, 82,… or 100 and repair-time equaled 3 h.

## 5. Computer Simulation Results and Discussion

Three types of schedules were generated to investigate the influence of estimation methods over the ability of achieving accurate predictions of failure-free time: Basic schedules, predictive schedules, and reactive ones. Solutions were searched for the first set of reliability characteristics: I = {*a* = 58, *MTTF* = 93, *b* = 102, *MTTR* = 3} and the second II = {*a* = 23, *MTTF* = 88, *b* = 125, *MTTR* = 3} and for changing real failure-free times.

First, the basic schedules were obtained for job shop scheduling problems: (11 × 10), (10 × 9), (9 × 8), (8 × 7), and (7 × 6) using the immune algorithm [21]. The immune algorithm was run six times for each case problem. The best basic schedules were obtained for the task sequences described in Table 4 using the objective function of Equation (42).

**Figure 3:**
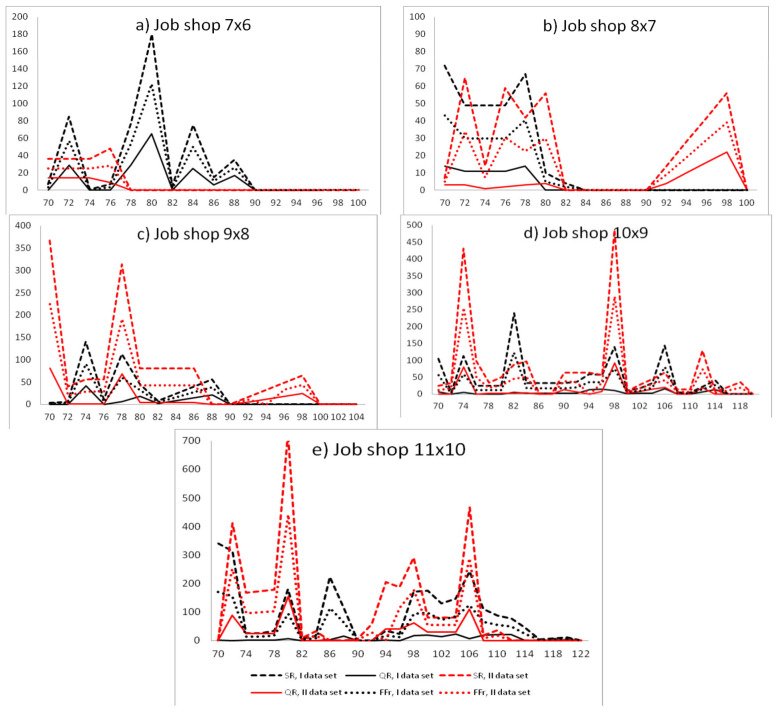
The impact of the critical machine failure time on the solution robustness (*SR*), quality robustness (*QR*) and weighted function (*FF*r) of *SR* and *QR* for scheduling problems for reliability characteristics obtained using the maximum likelihood method (dataset I) and empirical moments method (dataset II).

The MIDOS rule [21] was used to generate predictions for both sets of reliability characteristics (I, II). After a critical machine failure, two RSh and RDO reschedule heuristics were applied. The best reaction schedule was selected using Equation (51). The impact of the machine uptime on two criteria was examined: The solution robustness (SR) and the quality robustness (QR) (Figure 3). The x-axis represents the actual failure-free times of the machine, *X_m_*_+1,1_ = 70, 72, …, *E*, where *E* depends on the size of the scheduling problem and represents the machine end time. The y-axis represents values of criteria *SR* (48), *QR* (49), and *MIROS* (51) of reactive schedules, where the maximum value obtained for the quality robustness and solution robustness criterion equaled to 1, *QR*(u**) = 1, *SR*(u**) = 1.

Analyzing the impact of the machine failure time on the *SR* of predictive schedules generated for the first set of reliability input data for problem (7 × 6), one can notice the following phenomenon: The solution robustness of predictive schedules increased with failure-free operation of the machine until 80. After reaching the peak value, the solution robustness decreased with the failure-free time of the machine (Figure 3a). The same trends were noticeable for the quality robustness and weighted function *FF* (32) (Figure 3a). All failures happened before the predicted *MTTF* (equal 93).

Analyzing the instances of (8 × 7) and (9 × 8), the longer the failure-free time of the machine was, the better the solution robustness, quality robustness, and weighted function of the reactive schedule were (Figure 3b,c). Analyzing the instances of (10 × 9) and (10 × 11), when the real failure time approached the predicted value of *MTTF*, the better the solution robustness, quality robustness, and weighted function of reactive schedule were (Figure 3d,e).

The obtained schedules were also presented for the second set of reliability input data, for all instances of job shop problems (Figure 3). Analyzing the instance of (7 × 6), the longer the failure-free time of the machine was, the better the *SR*, *QR* and *FF* were (Figure 3a). Analyzing the instances of (8 × 7), (9 × 8), (10 × 9), and (10 × 11), when the real failure time approaches the predicted value of *MTTF*, the better the *SR*, *QR,* and *FF* of reactive schedules were (Figure 3b–e).

**Figure 4:**
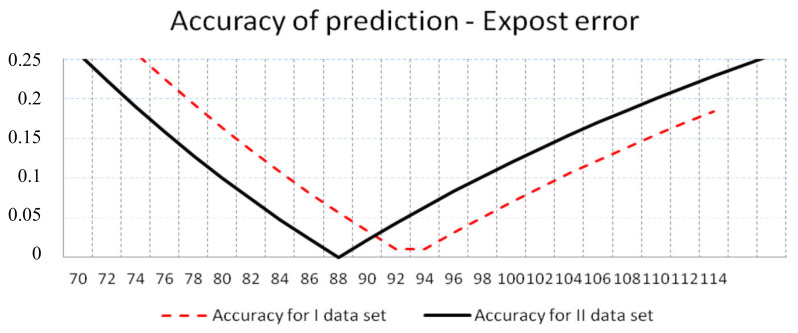
Accuracy of prediction of failure-free time obtained using the empirical moments approach.

In most instances, the phenomenon of achieving lower values of *SR*, *QR*, and *FF* before and after the predicted *MTTF* was noticeable. This proved that the truncated normal distribution was a useful distribution for failure time modeling. The higher the accuracy of failure-free time prediction was, the lower the values of the *SR*, *QR*, and *FF* were obtained. The best predictions were obtained for the value of accuracy less than or equal to 0.05; that is, for real failure times from 89 to 98 in the event of first dataset and from 84 to 93 in the event of second reliability dataset (Figure 4).

The span of normal distributions describing the failure-free time is very wide in both cases of reliability input data, I (*a* = 58, *b* = 102) and II (*a* = 23 and *b* = 125). It means that the *MIDOS* heuristic modifies the basic schedule extensively. In order to investigate the efficiency of the *MIDOS* rule application, one needs to examine the real failure times before and after the time span described by the safety period [*a*, *b* + *MTTR*], which is not the subject of this paper.

**Figure 5:**
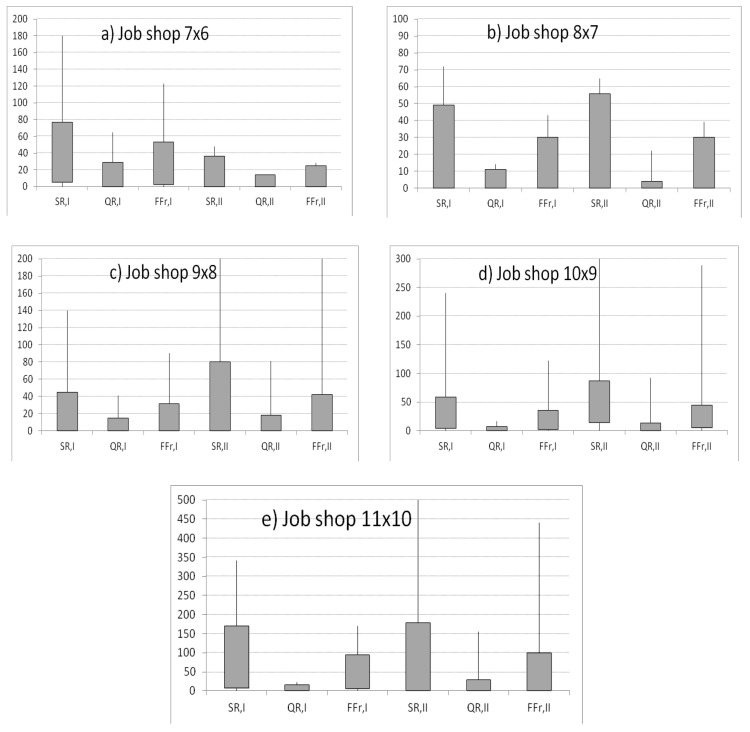
Reactive schedules assessed using solution robustness (*SR*), quality robustness (*QR*), and weighted function of *SR* and *QR* (*FF*r) for reliability input data anticipated using the maximum likelihood approach (dataset I) and empirical moments method (dataset II).

Let us analyze the maximal, minimal, first, and third quintile of the obtained schedules for the solution robustness, quality robustness, and weighted function for the two reliability input data (RID) sets. Analyzing the instance of (7 × 6), one can notice that the reactive schedules obtained for the second RID set absorbed machine disruptions more. The first and third quintiles equaled 0 and 25, respectively, for the weighted function *FF*. The first and third quintiles equaled 0 and 36, respectively, for the *SR* and 0 and 14 for *QR* (Figure 5a).

Analyzing the (8 × 7) instance, the quality of the reactive schedules obtained for first and second RID sets was equal. The first and third quintiles were equal 0 and 49, respectively, for *SR* for the I set of the RID. The first and third quintiles were equal 0 and 4, respectively, for the *QR* for the II set of RID (Figure 5b).

Analyzing the instances of (9 × 8), (10 × 9), and (11 × 10), the reactive schedules obtained for the I RID set absorbed machine disruptions more (Figure 5c–e). For example, for the (9 × 8) instance, the first and third quintiles were equal 0 and 31.5, respectively, for the weighted function *FF*.

Analyzing the results of the simulations, it could be stated that the method based on sample moments allowed for more accurate forecasting in the event of small-size job shop problems. The maximum likelihood approach allowed for more accurate predictions in the event of large-size scheduling problems.

### 5.1. Estimation of Reliability Characteristics for the Automotive Industry

A production line of electric steering gears was analyzed. Successive failure-free times were supposed to have truncated normal distributions and were followed by exponentially distributed times of repairs. Based on the data about the failure number and failure-free times of the critical machine, the following two reliability characteristics were calculated: Mean time to failure (*MTTF*), and period of increased probability of failure [*a*, *b* + *MTTR*]. The empirical moments approach was used to estimate the reliability characteristics.

The critical resource measured the average fractions of the cooperating components in the electric steering gear. Data on failure-free operation of the critical resource were collected from March to October 2019. Data on failure modes and maintenance actions taken for the resource of the final steering gear functionality test were also collected. The unknown failure modes resulted from the fact that the production line was in the “infant mortality period” of the life cycle and the maintenance staff was not experienced enough.

**Figure 6:**
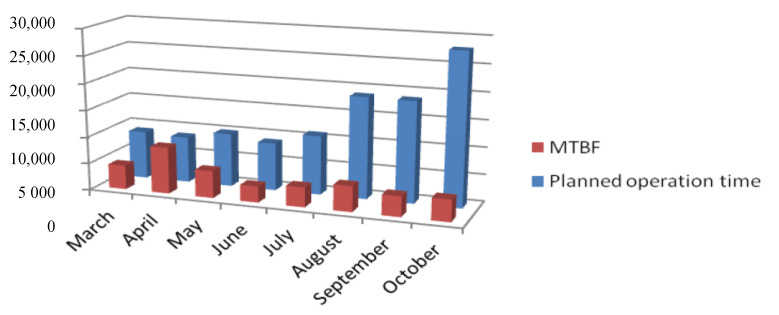
Impact of planned operation time of the station of final functionality.

The longest mean time between failures was in April. In April, there was only one failure; this was explained by the fact that the planned working time was relatively short, taking into account the entire period studied (Figure 6). In the following months, it was noticeable that the *MTBF* value stabilized, despite the increase in the planned working time and the monthly number of failures. The values of the *MTBF* varied from 61.33 to 78.75 h from July to October in 2019. The prediction of the failure-free time of the resource operation for December was needed.

Applying Equations (23) and (24) for the data on failure-free times, successive estimators of truncated normal distributions were obtained: *m*_1_ = 229.23, *m*_2_ = 0.06, *m*_3_ = 395.95, *m*_4_ = 170.79, *m*_5_ = 110.97, *m*_6_ = 199.81, *m*_7_ = 127.54, *m*_8_ = 104.80 h, and σ_1_ = 229, σ_1_ = 140.75, σ_2_ = 0, σ_3_ = 394.14, σ_4_ = 119.33, σ_5_ = 65.32, σ_6_ = 213.52, σ_7_ = 138.24, σ_8_ = 79.66 h. The values ${\hat{m}}_{9}$ and ${\hat{\sigma }}_{9}$ of the truncated normal distribution were predicted using the regression method. The trend function describing *m*_1_, …, *m*_8_ was f(*m*) = 4.3469⋅9^2^ + 28.624⋅9 + 149.44. The trend function describing *σ*_1_, …, *σ*_8_ was f(σ) = −6.3399⋅9^2^ + 53.103⋅9 + 66.579. The normal distribution truncated to the positive half-axis was described by parameters ${\hat{m}}_{9}$ =54.95 and ${\hat{\sigma }}_{9}$ = 30.97, and the expected value equaled to *EX* = 57.53 for December. The technical inspection of the resource was planned at time 57 h. The increased probability of the machine failure was indicated at the time window [42, 71 + M*TTR*].

## 6. Conclusions

A method of risk assessment and planning technical inspection of machines with optimization for planning production tasks based on the probability theory was presented. The aim was to develop a universal method which reflects the operation of the production system and the nature of the disturbances, allowing one to estimate unknown parameters regarding reliability. The frequency of the machine failures was described by the normal distribution truncated to the positive half-axis. Estimation methods of truncated normal distribution parameters were presented. The estimation methods were compared for different sized job shop scheduling problems. Predictive schedules were evaluated using the solution robustness and quality robustness criteria. The estimation method based on sample moments allowed us to obtain more accurate predictions in the case of small-sized job shop scheduling problems. The method based on maximum likelihood allowed for more accurate predictions in the case of large-sized job shop scheduling problems.

Future research will be continued for the production line of electric steering gears with more than one critical resource failing. A method for estimating reliability characteristics will be given for distributions depending on the phases of life cycles of machines. It is also planned to apply other methods of statistical estimation of distribution parameters. Inspired by [36], a model of electric steering gear production system with state transitions of stochastically dependent machines has been proposed. Machine degradation rates depend on operating conditions and deteriorate from a better state to worse one.

## Figures and Tables

**Table 1:** Failure-free times *x_i_*_,*k*_ of the critical machine collected for scheduling horizons, *i* = 1,2,…,35.

The Number of Failures of the Critical Machine (and Failure-Free Times) in Scheduling Horizon *i*				
9(90,90,100,110,100, 110,110,130,130)	7(100,105,105, 120,120,105,105)	9(110,90,105,120, 120,100,110,130,90)	6(105,120,140, 160,180,200)	10(90,130,135,90, 75,80,125,90,80,100)
11(100,90,95,95,90 ,90,85,85,90,90,80)	8(100,140,150, 140,180,100,105,80)	6(50,100,150,150, 280,270)	8(200,100,120, 130,150,90,100,90)	9(90,130,100,90, 130,120,130,90,100)
9(80,90,100,110,120, 125,120,100,110)	7(100,120,125,135, 140,150,160)	10(110,90,95,90,80, 100,90,100,90,90)	11(100,90,80,80, 85,80,90,90,85,70,70)	10(90,95,100,100, 90,150,80,90,80,80)
9(80,90,100,110,120, 125,120,120,130)	7(100,120,125,135, 140,140,160)	8(90,90,100,110,120, 120,140,160)	7(100,110,120, 145,155,150,160)	9(120,110,105,105, 110,100,100,110,120)
9(80,85,100,110,115, 110,120,120,140)	8(80,100,120,125,135, 140,140,150)	9(75,80,85,90,110, 120,130,140,150)	9(70,80,100,110, 115,120,135,130,140)	7(100,110,120, 130,140,140,180)
11(50,60,60,70,80,85, 100,110,115,120,125)	10(55,60,65,65,80,90, 110,120,140,145)	7(90,100,130,140, 150,160,170)	10(70,75,80,85,90, 95,100,110,120,120)	11(55,55,60,80,85,90, 90,100,120,130,130)
11(45,60,60,65,80,90, 100,110,115,135,135)	11(45,55,60,65,70,90, 90,110,120,120,150)	10(40,55,60,90,95, 90,110,125,135,155)	9(80,90,90,95,100, 110,125,135,155)	10(55,60,80,90,90, 90,105,125,135,140)

**Table 2:** Calculation of parameters ${\hat{m}}_{i}$, ${\hat{\sigma }}_{i}$ and reliability characteristic mean time to failure (*MTTF).*

	$\mathrm{The}\;\mathrm{Prediction}\;\mathrm{of}\;{\hat{\sigma }}_{m+1}$ $,\;{\hat{m}}_{m+1}\;\mathrm{and}\;\mathrm{MTTF}\mathrm{Using}\;\mathrm{the}\;\mathrm{Gauss-Newton}\;\mathrm{Method}$			
	Maximum Likelihood Approach		Empirical Moments Approach	
${\hat{m}}_{36}$	y = 95.70 + 0.429x − 0.023x^2^	80.18	y = 99.25 − 0.988x + 0.009x^2^	76.39
${\hat{\sigma }}_{36}$	y = 52.27 + 0.09x − 0.023x^2^	44.38	y = 63.27 − 1.464x + 0.028x^2^	47.22
The Prediction of ${\hat{\sigma }}_{m+1}$, ${\hat{m}}_{m+1}$, and *MTTF* Using the Least Squares Method				
	Maximum Likelihood Approach		Empirical Moments Approach	
${\hat{m}}_{36}$	y = −0.0239x^2^ + 0.4291x + 95.705	80.17	y = 0.0103x^2^ – 1.0867x + 99.906	74.13
${\hat{\sigma }}_{36}$	y = $0.001x^{8\cdot 10^{-15}}$	44.41	y =0.0281x^2^ – 1.4997x + 63.531	45.95

**Table 3:** Evaluation of the estimated models achieved using the Gauss–Newton method with a maximal number of iterations: 50.

$\hat{m}$ Estimated Using the Maximum Likelihood Approach							
	Coefficient	SE	*t* Statistic	R^2^	*p*-Value	95% Confidence Interval (Left End)	95% Confidence Interval (Right End)
a0	95.70475	8.430837	11.35175	0.095	0.000000	78.53170	112.8778
a1	0.42915	1.079850	0.39741		0.693702	−1.77044	2.6287
a2	−0.02390	0.029096	−0.82125		0.417581	−0.08316	0.0354
$\hat{\sigma }$ Estimated using the maximum likelihood approach							
a0	52.27978	6.464445	8.087281	0.040	0.000000	39.11214	65.44743
a1	0.09115	0.827988	0.110085		0.913030	−1.59541	1.77771
a2	−0.00862	0.022310	−0.386394		0.701762	−0.05406	0.03682
$\hat{m}$ Estimated using the empirical moments approach							
a0	99.25615	12.08471	8.213366	0.083	0.000000	74.64040	123.8719
a1	−0.98821	1.54785	−0.638439		0.527731	−4.14108	2.1647
a2	0.00981	0.04171	0.235257		0.815508	−0.07514	0.0948
$\hat{\sigma }$ Estimated using the empirical moments approach							
a0	63.27331	11.33485	5.58219	0.06209691	0.000004	40.18497	86.36164
a1	−1.46463	1.45181	−1.00883		0.320622	−4.42186	1.49260
a2	0.02830	0.03912	0.72350		0.474628	−0.05138	0.10798

**Table 4:** The quality of best basic schedules obtained for job shop problems using the MOIA (Multi-objective immune algorithm).

The Size of the Problem	The Job Sequence	*Cmax*	*F*	*T*	*I*	*FFy*
7 × 6	{5 2 3 4 6 0 1}	117	273	330	0	1.02
8 × 7	{5 7 2 3 4 0 6 1}	126	335	434	0	1.02
9 × 8	{8 5 2 3 0 4 7 6 1}	137	415	553	0	1.01
10 × 9	{8 5 2 3 4 6 9 0 7 1}	164	530	756	0	0.99
11 × 10	{3 8 2 5 4 7 6 9 0 1 10}	169	623	863	0	1.05
